# A bibliometric study of social isolation and biomarkers of ageing

**DOI:** 10.1007/s44192-026-00563-9

**Published:** 2026-08-07

**Authors:** Navya Sankeerthana CH, Dipsikha Sahoo, Maneshwar Thakur

**Affiliations:** 1 Department of Applied Psychology, GITAM (Deemed to be) University, Hyderabad, Telangana 502329 India; 2 Department of Sociology, GITAM (Deemed to be) University, Hyderabad, Telangana 502329 India; 3 Department of Social Work, Central University of Karnataka, Kalaburagi, Karnataka 585367 India

**Keywords:** Social isolation, Biomarkers of ageing, Bibliometric analysis, Performance analysis, Thematic evolution

## Abstract

Research on social isolation suggests that psychosocial factors are not only associated with adverse mental health conditions but also associated with various neurobiological and immunological processes. The physiological mechanisms underlying the health effects of social isolation can be indicated by various biomarkers such as cortisol, cytokines, C-reactive protein, telomere length, DNA methylation, epigenetic clocks. Though the research concerning the relationship between social isolation and biomarkers of ageing is expanding, a detailed and comprehensive picture of global research trends, emerging patterns of themes is limited. The present study attempts to examine the scientific production, influential contributions of authors, evolution of major research themes on social isolation and biomarkers of ageing. Scopus database has been used to retrieve relevant publications using a structured search strategy. Systematic and transparent data extraction, filtering, and cleaning procedures resulted in construction of the final bibliometric dataset. The records were published during the time period of 1971–2026 and included only scientific articles, review and English-language documents. The final dataset consisted of 1973 publications. Bibliometrix packages in Rstudio are used to conduct performance and conceptual mapping. The findings of the study are represented under performance indicators, thematic mapping, evolution of themes during different time slices and factorial analysis. The results reflected the major contributions from developed countries and leading institutions showing scholarly interest in the biological consequences of social isolation. The future research can be directed towards finding relationships between biopsychosocial determinants of overall well-being of the person, developing intervention strategies aimed at reducing the negative consequences of social isolation.

## Introduction

Social isolation, an underrated public health risk, has recently emerged as a challenge among the ageing population. The paper provides a systematic, quantitative foundation to map the research trends in social isolation and its impact on biomarkers of ageing and the interdisciplinary nexus of gerontology, biomarkers research, and social epidemiology.

“Man is a social animal”, a famous statement by Aristotle, highlights the undeniable importance of human society for human survival. The behavioural, physiological, neurological, and genetic mechanisms of human evolution have been sustained by social engagement. The need for interpersonal interaction, belongingness, and emotional connection is significant for both physical and psychological well-being. Social isolation involves an objective feeling of lack of social contact and a subjective sense of feeling alone (perceived social isolation), making it multilayered and complex [1, 2]. Social isolation has been a significant factor in determining both physical and mental health outcomes in recent decades. According to Need to Belong theory, individuals have an innate desire to establish and maintain interpersonal connections [3]. According to Pancani et al. [4], consistent and constructive social interactions are crucial for mental well-being. A lack of social ties may lead to emotional distress and lower life satisfaction, affecting physiological health.

Much like physical pain or hunger, social isolation is a neurobiological indication of social threat, which may lead to a fight for survival. It triggers insecurity, hyper vigilance for dangers in the environment, and motivation to reestablish connectivity. Implicit constant monitoring of the environment alters the physiological mechanisms of the body, resulting in poor quality of sleep, raising morbidity and death (Loneliness model, [5]). From a sociological perspective, social isolation is more than an individual condition, but is determined by social structures and institutions. A study by Zhang et al. [6] reveals that people who are integrated into the communities have lower levels of anxiety, sadness, and behavioural issues.

Research has shown that social ties promote physical health by encouraging positive lifestyle habits such as diet, exercise, quitting smoking and alcohol, and better adherence to treatment plans [7, 8]. Uchino [9] finds that social support has a positive influence on cardiovascular health of individuals which is an outcome of psychological processes like mood, sense of control, and consistency in practising health behaviours. Social isolation can increase the symptoms of anxiety, depression and in many cases, suicidal ideation among adolescents as well, apart from ageing individuals [10]. A systematic review of 63 research studies uncovered a significant correlation between loneliness and mental health issues [11]

The paper by Cudjoe [12] examined the association between social isolation and Interleukin (IL-6) and C-reactive protein. The study defined social isolation in terms of living arrangement, community network, social participation in religious gatherings. The findings showed that individuals who did not participate in the social activities have high activity in the inflammatory pathways of IL-6 and CRP, linking psychosocial stress to clinical outcomes.

Braig [13] examined the influence of social isolation and loneliness on physical health and death risk in 1459 older adults (65 + years old) from a German community. High social isolation (SI), especially from friends, was linked to slightly elevated biomarkers of inflammation (hs-CRP), heart stress (GDF-15, hs-cTnT), and cardiac function (NT-proBNP) over three years. He concluded that social isolation can also reduce bodily movement and physical strength which increases the mortality risk by 10-years when compared to individuals who are active in their social circles. The studies on how social isolation is linked to structural and functional difficulties of the brain has been another emerging area of research. A study by Lammer et al. [14] found the reduced volumes of hippocampus and cortical thickness is associated with lonely individuals. Another similar work by NJ and Jeffery [8] also identified cognitive impairments like memory deficit and reduced attention span among older people who live alone.

Biomarkers are quantifiable bio-indicators that show how physiological processes are associated with psychosocial conditions. The field of ‘Biomarker’ is advancing the research frontier, elucidating the influence of the social environment on both psychological and physiological health of individuals. Since these are objectively measurable, it could be analysed how social experiences and psychological states become embedded in the body. For example, the immune system, hormonal fluctuations, and inflammation within the vital organs could be largely influenced by prolonged psychosocial stress, social isolation, and loneliness [15]. The biomarkers specifically related to biological ageing are used by researchers in social epidemiology and psychology to find out the risk of disease in different age groups [16]. Some significant biomarkers like cortisol levels, C-reactive protein (CRP), interleukin-6 (IL-6), telomere length, and epigenetic clock play a major role in understanding how psychosocial distress can impact ageing by triggering bodily responses [17].

### Hypothalamic–pituitary–adrenal axis

The hypothalamic–pituitary–adrenal (HPA) axis which is measured by cortisol appears to be a potential biomarker of ageing. Research reveals that this axis is sensitive to social isolation due to its role in stress response via cortisol regulation. A research study conducted by Brandt et al. [1] also reveals that HPA axis gets activated by social isolation and loneliness resulting in rise of basal cortisol, flattens diurnal slopes, particularly in older persons. Few studies suggest that social connectivity reduces the activity of the HPA axis leading to inhibitory effect in acute psycho-emotional distress [18, 19]. Though loneliness and social isolation are major psychosocial stressors, dysregulation of the HPA axis typically depends on age, duration and context, and it leads to altered cortisol rhythms [20, 21]. Hence research evidence suggests that absence of meaningful social relationships can result in sustained HPA activation, resulting in physiological decline and hastens biological ageing.

### Inflammation markers

Research on cytokines suggests that isolation triggers sympathetic nervous system leading to activation of cytokines which might result in cardiovascular diseases, depression and cognitive decline. Inflammation markers can be traced in blood or other fluids that reflect activation of the immune system. They help to find presence, severity, and at times prognosis of acute and chronic diseases. IL-1β, IL-6, TNF- α and IFN-γ are pro-inflammatory cytokines which can be activated by social and environmental triggers [22]. Another important researcher Cole [23]explained this process as Conserved Transcriptional Response to Adversity (CTRA).

### Telomere length (TL)

Telomere Length (TL) is a measurement of chromosome ends that shortens with cell divisions and contributes to cellular ageing. Neurodegenerative diseases like Alzheimer's, cardiovascular disease and other medical conditions like high blood pressure are often associated with shorter leukocyte telomeres. Research conducted by Srinivas et al. [24] found that genetically longer telomeres can increase cancer risk and they are influenced by environmental factors as well. TL may mediate the adverse effects of gene expression even in the case of genetically inherited diseases [25].

### Epigenetic ageing

The social isolation elevates biomarkers which dysregulate stress hormones such as cortisol, accelerate telomere shortening, and hasten epigenetic ageing via DNA methylation clocks. Epigenetic ageing also reflects the biological validity of cellular senescence, mitochondrial dysfunction, metabolic dysregulation, associated with adverse physical and psychological health consequences. The chronic low-grade inflammation can be triggered by psychosocial stressors like loneliness and social isolation which can be captured by epigenetic clocks at molecular level [26]. Research studies support a consistent link between social isolation/loneliness and accelerated DNA methylation across population cohorts as well [27, 28]. A study conducted by [27] measured epigenetic age acceleration among middle aged adults in the US. The results revealed that loneliness amplified negative health consequences resulting in changes of their DNA methylation. The study also found that epigenetic age assessments and findings of loneliness tests predicted chronic conditions like multimorbidity using GrimAge clock, Horvath, DunedinPACE. The research evidence inclines towards showing strong association between social connectivity, wider network of family, friends and lower pace of molecular ageing.

The abovementioned research evidence provides information on how social isolation influences biological ageing, immunological responses, stress management, and molecular ageing processes. The present study has considered a few of the biomarkers namely, cortisol, telomere length, inflammatory markers (CRP, IL-6) and epigenetic clocks. Social connectivity, community life, and interpersonal relationships lower the risk of chronic inflammation, reduce activation in the HPA axis and also slows down cellular ageing. Hence, this bibliometric analysis of social isolation and biomarkers of ageing attempts to understand the research trends, theoretical and empirical gaps, and future implications of how psychosocial factors would impact ageing at different physiological parameters.

Though there are empirical studies and systematic reviews on biomarkers of ageing, they lack a comprehensive assessment of scientometrics in understanding the conceptual and social dimensions. The present work tries to fill this gap by quantifying research productivity, citation dynamics, collaboration works between authors, evolution of relevant themes and identifying the future research trends. Though in the existing literature, there are systematic reviews and empirical studies on social isolation and biomarkers of ageing, a bibliometric study combining the intellectual structure or trend analysis on social isolation and biomarkers of ageing is currently not present in the published literature.

The present study aims to provide an overall framework of the social isolation and biomarkers of ageing research landscape, highlight global collaborations, and identify the emerging research trends, such as the importance of psychosocial factors in influencing neurobiological processes of ageing. By trying to build a connection between psychology, physiology and neurobiology, this paper contributes to the interdisciplinary nature of knowledge output in the mental health research area. The findings can be significant for researchers, clinicians, public health professionals, organisations working with elderly population as it tries to explain biological ageing as one of the outcomes of social isolation.

The main objectives of the bibliometric study on social isolation and biomarkers of ageing are: firstly, to find out and analyse the annual publication trends, most influential sources, authors, documents and institutional affiliations. Secondly, the paper aims to identify the country-wise distribution and international collaborations over time. Thirdly, it seeks to understand the key thematic conceptual clusters and themes emerged across different time periods (1971–2026). Fourthly, it attempts to explain the trajectories of research traditions with conceptual gaps that characterise the current body of literature on social isolation and biomarkers of ageing. Fifthly, to identify the gaps in the existing research literature to direct further studies in similar fields.

## Data and methodology

**Fig. 1:**
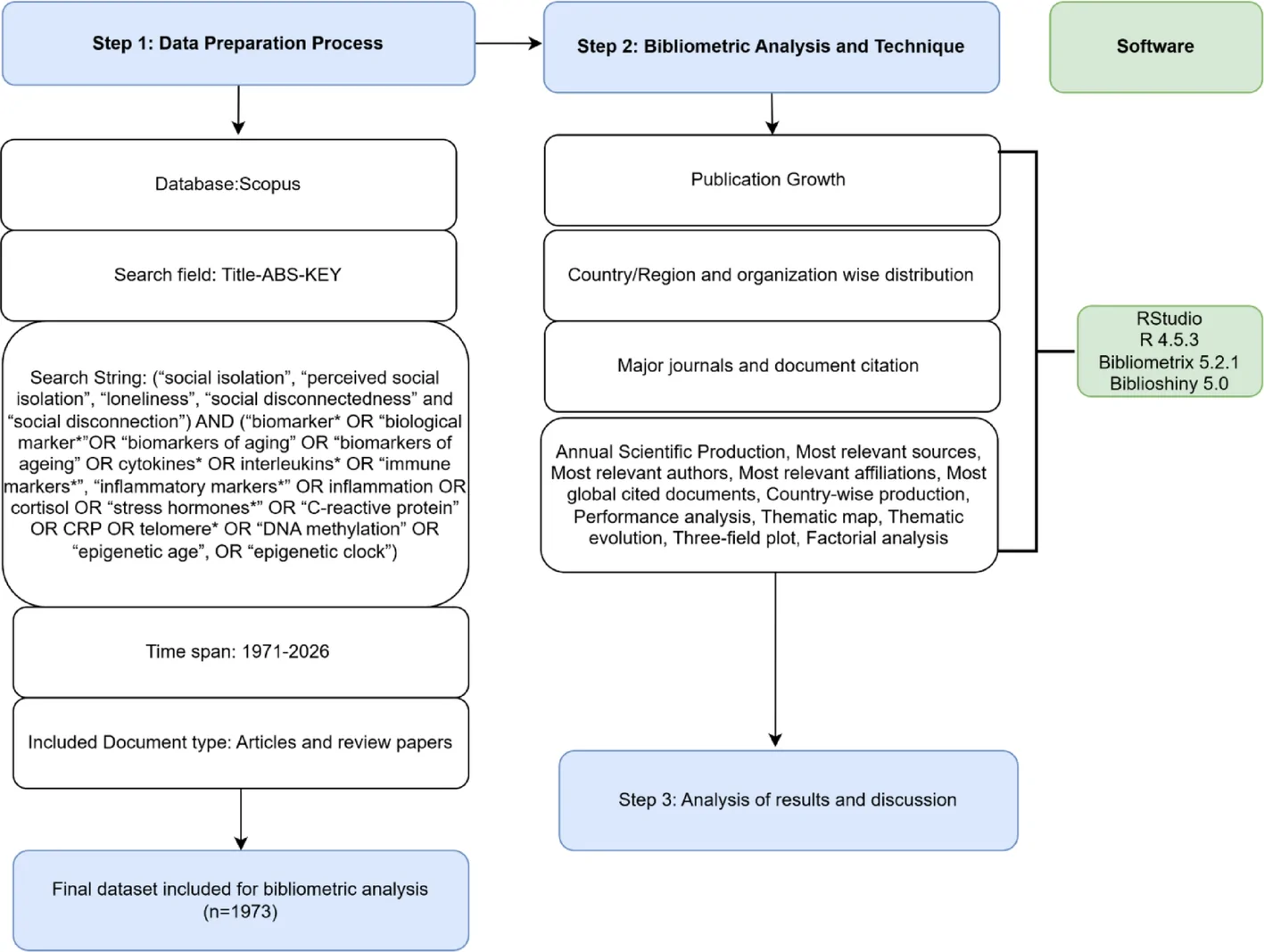
Framework of the bibliometric research design and analytical procedure

The current work of bibliometric study employs a systematic and transparent approach to examine the research landscape relevant to social isolation and biomarkers of ageing. The publications were retrieved from the predefined search strings on the Scopus database and filtered according to the inclusion and exclusion criteria. The resulting dataset was further subjected to the bibliometric analysis, using the Bibliometrix package of RStudio and its web-interface, Biblioshiny [29]. The bibliometric techniques facilitated comprehensive performance analysis and conceptual mapping of publications. The data were extracted, analyzed and examined the publication trends, patterns of collaborations, thematic mapping and thematic evolution over three different periods of time. Hence this bibliometric assessment aims to identify the major research themes, influential authors, collaboration networks and evolving knowledge structure of research on social isolation and biomarkers of ageing during the time period of 1971–2026. Figure 1 presents the overall bibliometric research design adopted in this study, including data retrieval, dataset preparation, analytical procedures, and interpretation of results.

### Data sources and search strategy

The Scopus database was used for retrieving the research publications on 18th March, 2026 to ensure the inclusion of the most recent publications. Scopus is one of the largest multidisciplinary citation databases which covers peer-reviewed literature across several disciplines making it suitable for interdisciplinary topics like social isolation and biological ageing. The Scopus database is preferred because of its broad coverage of multidisciplinary studies. It also provides a comprehensive citation indexing, which is suitable for bibliometric analysis. Using this as a single database ensures consistency in indexing standards, metadata extraction, citation counts, h-index, co-citation network, and potentially reduces duplication. Scopus is known to have extensive coverage of social science research [30] Additionally, Scopus database has been frequently used in bibliometric research papers because of its extensive standardized metadata and compatibility with software tools of R-studio and Biblioshiny [31, 32].

The search strategy consisted of keywords related to social isolation and biological markers of ageing. The keyword groups were combined using Boolean operators to maximize the number of studies relevant to the study. The SPIDER (Sample, Phenomenon of Interest, Design, Evaluation, Research type) framework applied for the conceptual development of the search string. The individual groups examined in the studies of social isolation and its related constructs like loneliness and social disconnection make up the sample (S). The phenomenon of Interest (PI), comprises social isolation, loneliness, social disconnection. Design component (D) includes the relationship between biological indicators of ageing and social isolation and how they are studied in different research works. Evaluation (E) includes the biomarkers such as cytokines, interleukins, C-reactive protein, cortisol, telomere length, DNA methylation, and epigenetic ageing which can be broadly categorised as biological markers of ageing. The research type (R) consists of relevant studies which are of quantitative, qualitative and mixed-methods designs, indexed in the Scopus database.

The first group consisted of terms “social isolation”, “perceived social isolation”, “loneliness”, “social disconnectedness” and “social disconnection”. This string was applied in the fields of title, abstract and keywords in the Scopus database. The search has resulted in a total 88,538 records.

The second group of keywords included terms like “biomarker”, “biological marker”, “biomarkers of ageing”, “biomarkers of ageing”, “cytokines”, “interleukins”, “immune markers”, “inflammatory markers”, “inflammation”, “cortisol”, “stress hormones”, “C-reactive protein (CRP)”, “telomere”, “DNA methylation”, “epigenetic age”, and “epigenetic clock”. The search has resulted in 3,352,013 records when applied independently.

Further these two groups of keywords were combined to extract research publications related to social isolation and biological markers of ageing. This has produced 2330 records from the database. The retrieved data were found between the time span of 1971 and 2026. Following pre-defined filters on scopus were applied: the document types and non-English articles. Filtrations such as removal of document types and non-English publications have excluded 354 records and arrived at 1976 results. The filtered dataset had been exported in BibTex format for further removal of duplicate records on Zotero. Three duplicate records were manually identified and merged using Zotero (ver. 8.0.4). Zotero’s duplicate detection feature was used to find duplicate records, which were then manually reviewed. Using Zotero’s merge interface, bibliographic attributes such as item type, title, author(s), publishing source, publisher, publication year, volume, page numbers, journal abbreviation, DOI, URL, ISSN, archive information, and language were compared for each possible duplicate set. Redundant entries were combined in accordance with the most accurate and comprehensive record, which was kept as the master item.

A final data set of 1973 publications is exported and analyzed using the Bibliometrix package (version 5.2.1) and its web-based interface Biblioshiny 5.0 within the R environment (version 4.5.3). After the dataset retrieval, filtering, and cleaning procedures, several bibliometric techniques like performance analysis, thematic mapping, thematic evolution analysis, three field-plot and factorial analysis were applied.

#### Inclusion and exclusion criteria

All the information such as citation details, bibliographical information, abstract and keywords were downloaded from the Scopus database. Two types of publications were included for the analysis: articles and review on social isolation and biomarkers of ageing. Editorials, notes, letters, book chapters, conference papers, short surveys, erratum, retracted publication and books were excluded because they may not reflect the same level of scholarly peer review, methodological rigour and lack consistency with respect to dataset, indexing and citation. Scientific articles and reviews in English were limited under the category of source type.

#### Data selection procedure

**Fig. 2:**
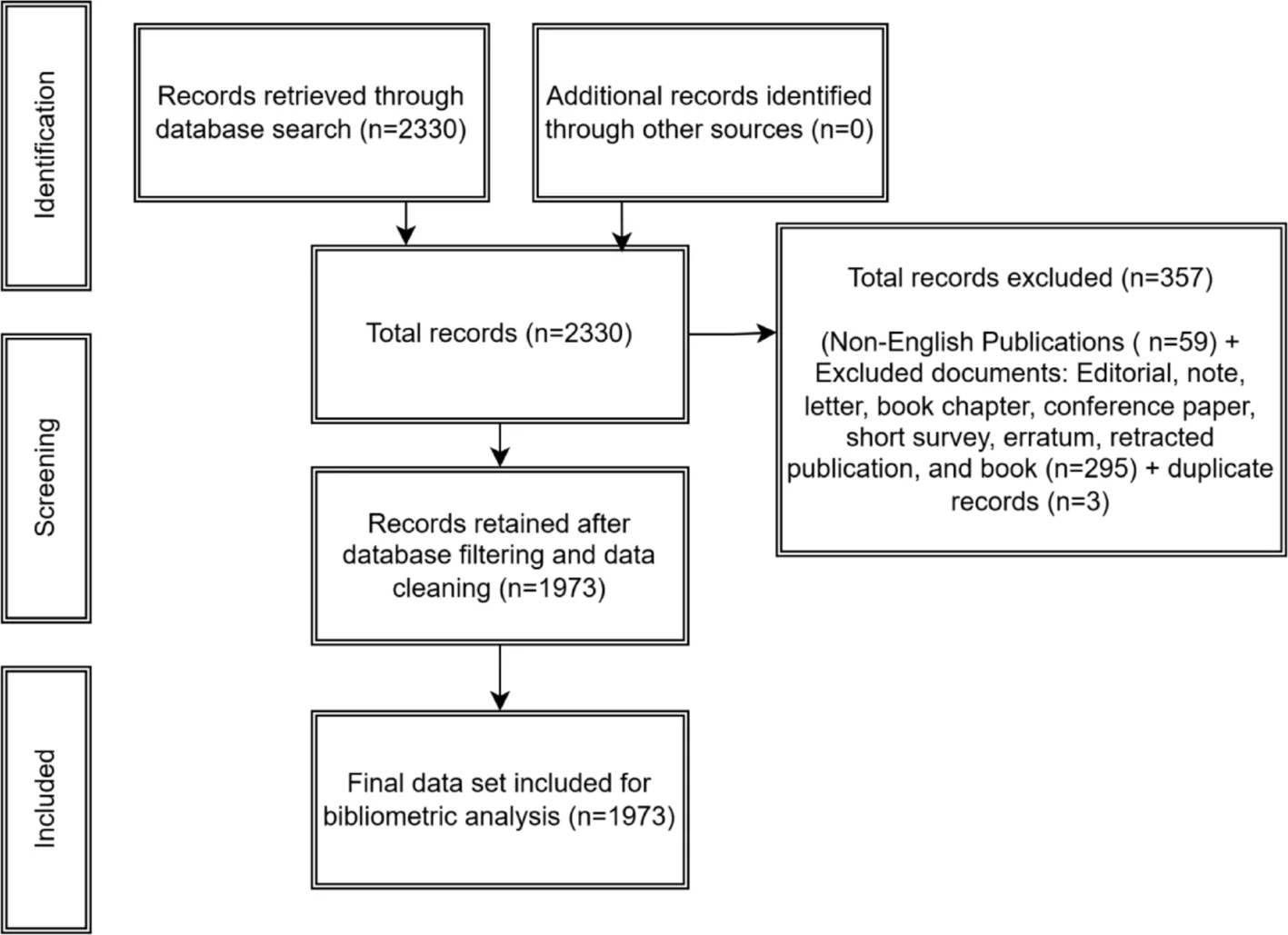
Flow diagram of dataset identification, filtering, duplicate management, data cleaning, and inclusion process

The workflow used for dataset identification, database-level filtering, duplicate management, and final dataset construction for bibliometric analysis is illustrated in Fig. 2. A total of 2330 records were retrieved from the initial search on the Scopus database. The database filtering and data-cleaning process resulted in a final dataset of 1973 publications for bibliometric analysis.

### Bibliometric analysis and analytical technique

Bibliometrix package of RStudio is used to illustrate the following analysis in images and visual representation of research publication trends of social isolation and biomarkers of ageing:

#### Performance analysis

Annual publication trends, leading journals, most cited authors, institutional affiliations, citation impact are recorded.

#### Thematic map

Thematic mapping was conducted using Author Keywords (DE) as a unit of analysis to identify and classify the major research themes within the field. For thematic mapping, the number of words was set at 100 and the minimum cluster frequency at 10. The Louvain clustering algorithm was employed to identify thematic clusters. The analysis is based on Callon’s centrality and density measures, which evaluate the importance and development of thematic clusters within a research domain [29, 33]. Centrality reflects the degree of interaction between a theme and other themes in the research field, whereas density indicates the internal cohesion and level of development of a thematic cluster [33]. Based on these dimensions, themes are classified into motor themes, niche themes, basic themes, and emerging or declining themes [32, 33].

#### Thematic evolution analysis

Based on the technique applied in the thematic map on classifying research themes in different quadrants, thematic evolution examined the emergence of research themes in three different time slices- 1971–2005, 2006–2015, and 2016–2025 of how research concepts and trends shifted across different periods. The analysis was performed using Author Keywords (DE) as a unit of analysis and the number of words was set at 150 words, a minimum cluster frequency of 10, an inclusion index weighted by word occurrences, and a minimum inclusion index threshold of 0.2.

#### Three-field plot

The three-field plot visualized the relationships among influential authors, Author’s keywords, and publication sources, thereby illustrating the structural connections within the research field.

#### Factorial analysis

Multiple Correspondence Analysis (MCA) based on all keywords was employed to explore the conceptual structure of the field by examining relationships among the 50 most frequently occurring keywords [29].

## Results

### Publication trend

The study employs a comprehensive bibliometric analysis to examine the publication trend in this field. Key indicators of performance analysis include annual increase in publications, relevant sources, authors, affiliations, most globally cited documents, country wise production, and country-wise collaborations. Line graph, bar diagrams, and chord maps are used to present the data.

The following Table 1 presents the total number of 1973 documents retrieved from 907 sources within the time span of 1971–2026. The total number of authors is 9758. However, the annual increase in publications is 7.76 per cent, relevant sources and authors, affiliations, most globally cited documents, country-wise production, country-wise collaborations.

**Table 1:** A complete overview of the selected 1973 documents

Timespan	Source	Documents	Annual Growth rate
1971:2026	907	1973	7.76%
Authors	Authors of single-authored documents	International Co-authorship	CO-Authors per Document
9758	111	24.02	6.02
Author’s keywords (DE)	References	Document Average Age	Average citations per doc
4406	0	8.73	50.56

**Fig. 3:**
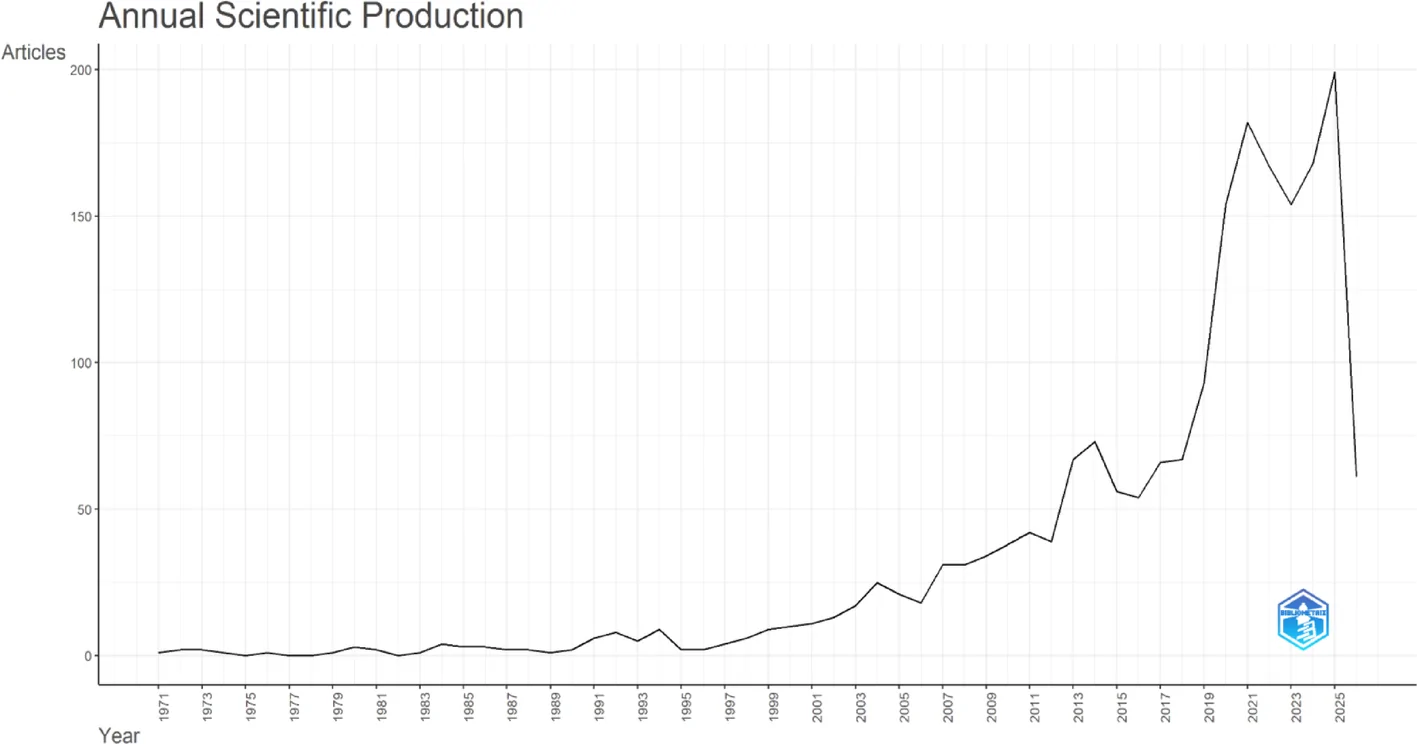
Annual scientific production

Figure 3 shows a growth in research output in the field from 1971 to 2025. In the initial years from 1971 to 1989, publication output remained low, indicating that the field was in an incipient stage. During 1990 and 2005, a gradual increase was observed, reflecting growing scholarly interest and conceptual development. The field entered a rapid growth phase, with a sharp increase in publications from 2006 onwards, although minor fluctuations are observed. After 2016, the most remarkable surge happened peaking around 2023–2024 (150–200 articles per annum).

A noticeable declining trend in 2025 is apparent, not due to an actual decrease in research activity but due to incomplete data for the current year. However, this figure indicates that this domain of research grew as a relevant topic in global health contexts, recognising health implications of social isolation in biomarker research.

**Fig. 4:**
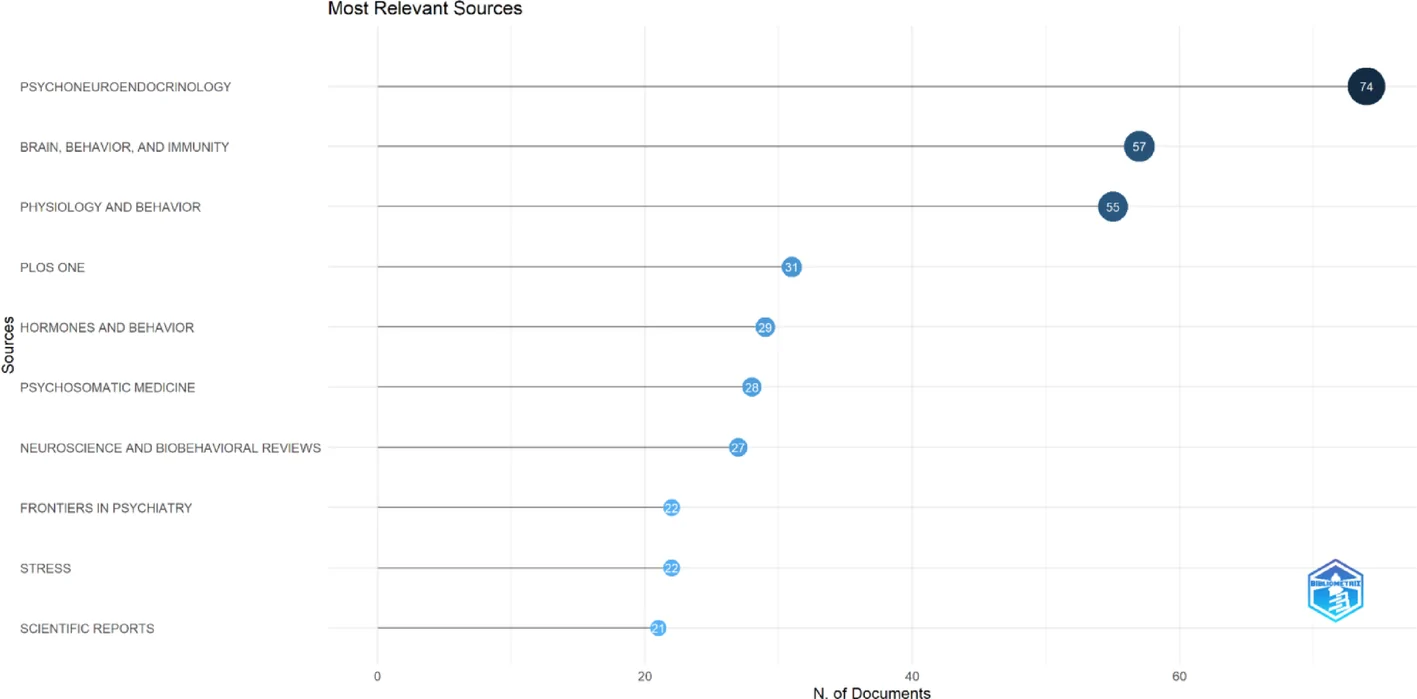
Most relevant sources

The ranking of the most relevant sources was made on social isolation and the biomarkers of ageing based on the total citation (TC) as presented in Fig. 4.

As per Fig. 4, *Psychoneuroendocrinology* ranks first with 74 publications amongst the core journals contributing to the field, followed by *Brain, Behavior, and Immunity* with 57, and *Physiology and Behavior* with 55. The mid-tier contributors include *PLOS ONE* (31), *Hormones and Behavior* (29) and *Psychosomatic Medicine* (28). This finding can be read in conjunction with Table 1 to understand that the journals with the highest publication volume do not necessarily correspond to those highly cited research articles.

**Fig. 5:**
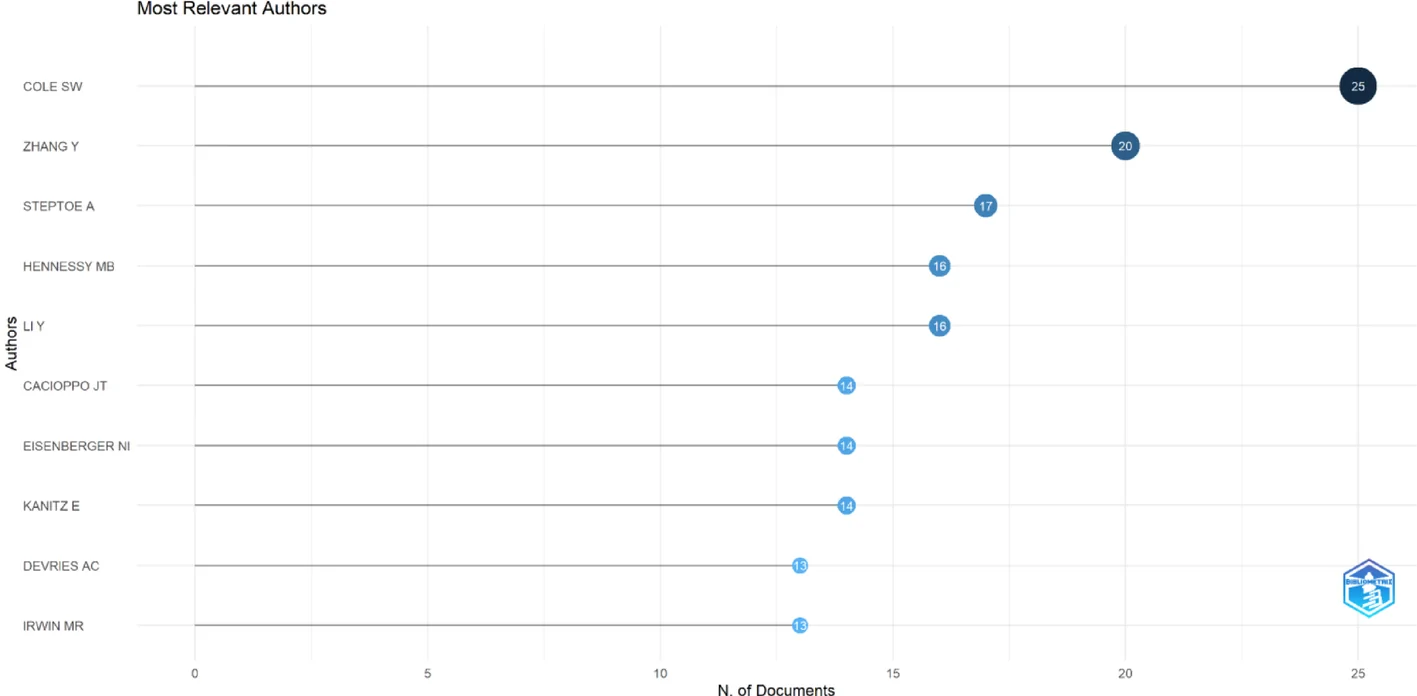
Most relevant authors

The following Fig. 5 highlights the authors with the most number of publications in the field. The authors who contributed to this field are Cole SW (25 publications), Zhang Y (20 publications), Steptoe A (17 publications), Hennessy MB and Li Y (16 each) and Cacioppo JT, Eisenberger NI, and Kanitz E (14 each).

The scholarly contributions of John T. Cacioppo and Naomi I. Eisenberger emphasise the importance of social neuroscience and stress research in the field of social isolation and biomarkers of ageing. These authors work on loneliness, stress responses, and neurobiological mechanisms, indicating the conceptual backbone of the field.

**Fig. 6:**
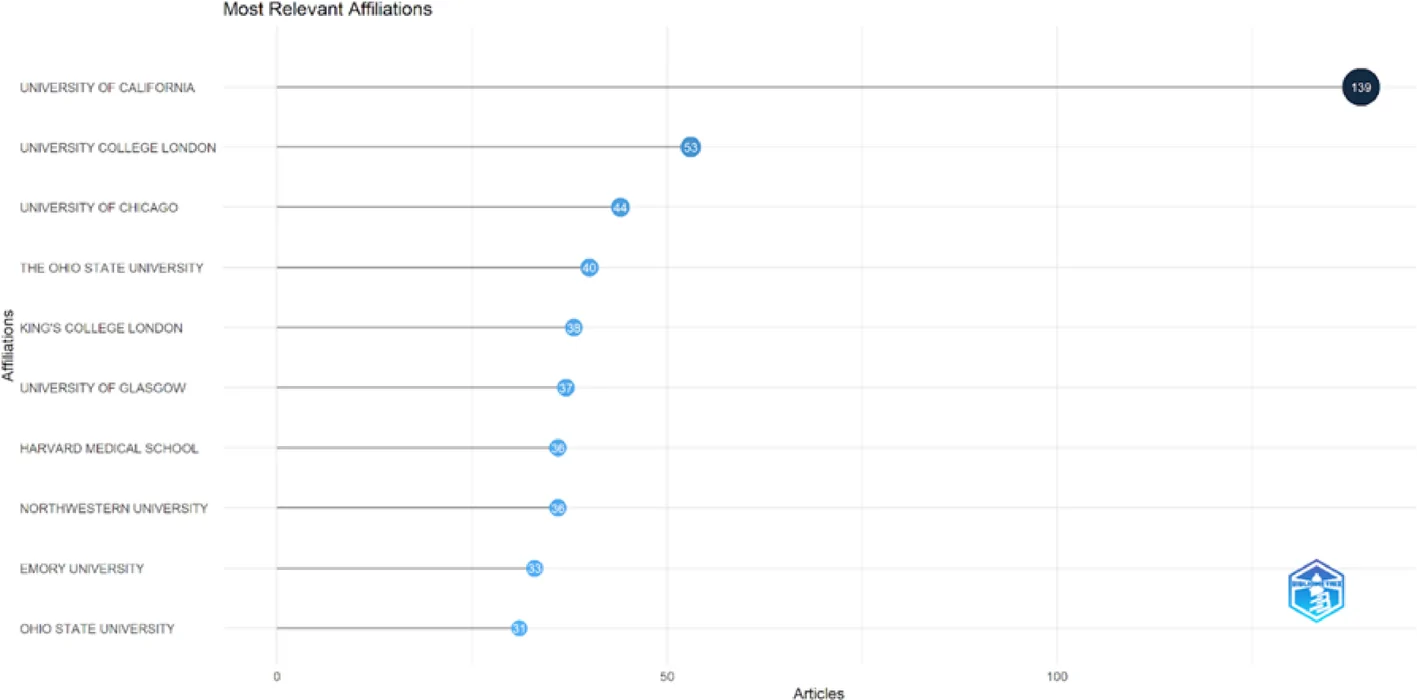
Most relevant affiliations

Figure 6 depicts those institutions that play a significant role in creating strong research hubs in Western countries. From 1971 to 2026, University of California led with 139 publications, followed by University College London with 53 in the second position and University of Chicago with 44 in third place. The publications indicate the presence of high-resource academic institutions in the US and UK.

Citation analysis identifies the most influential works shaping the field by showing the number of times the paper has been referenced by other researchers. The most cited paper is “A network theory of mental disorders”by Borsboom (2017) published in the journal of World Psychiatry (2496 citations) followed by “Dementia prevention, intervention, and care by Livingston et al. [34] published in the journal of Lancet (2057 citations); the third most cited document is “Allostasis and allostatic load: Implications for neuropsychopharmacology” by McEwen (2000) published in Neuropsychopharmacology (1450 citations). Research studies by Cacioppo (2015) and Danese (2009) are other more cited works.

**Fig. 7:**
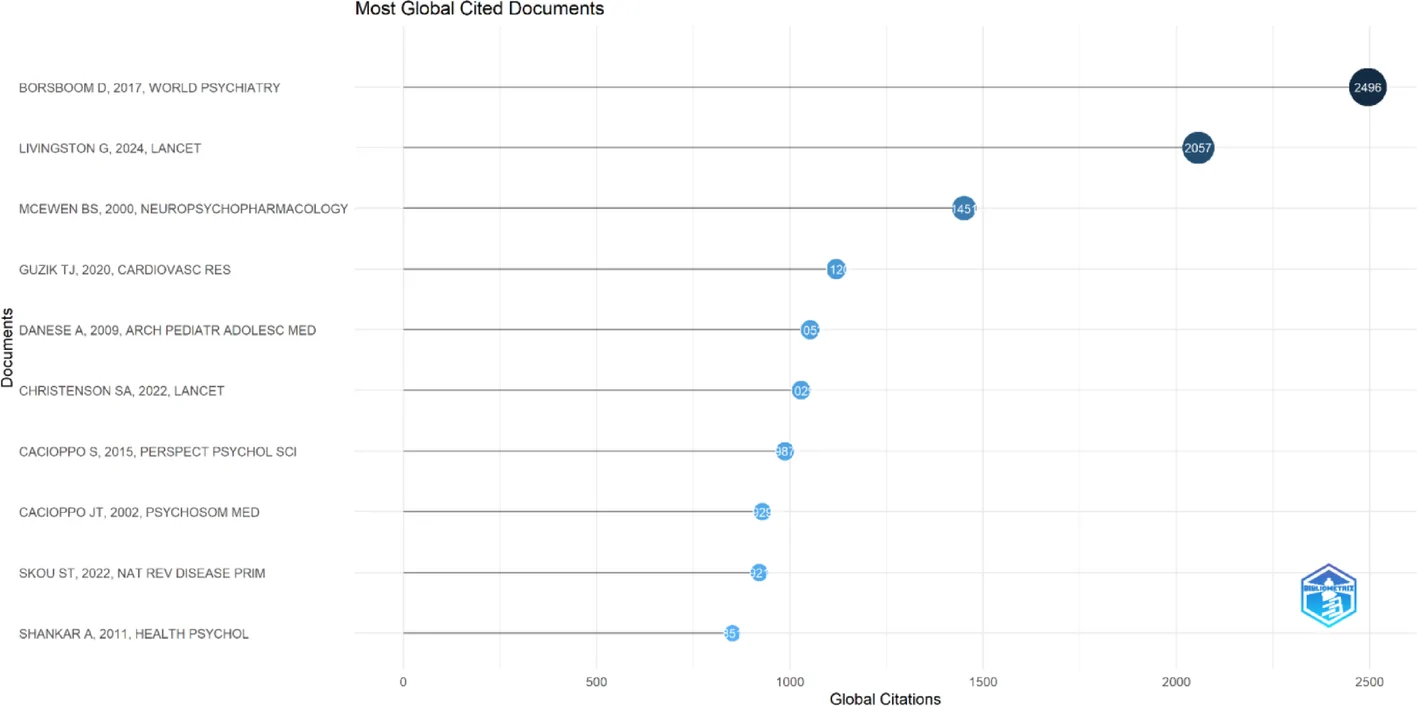
Most globally cited documents

Figure 7 also shows that McEwen’s work is mainly centred around stress biology and allostatic load. The highly cited recent studies (Lancet) show the effect of global disease burden and its relevance to public health. Hence, it suggests a strong conceptual foundation with its evolving nature.

Table 2 lists out ten most cited documents in this area of research. The paper by Borsboom published in World Psychiatry has the highest citation (2496) followed by Lancet (2057) and Neuropsychopharmacology (1451).

**Table 2:** Most cited documents

Paper	DOI	TC	TC Per year	Normalised TC
BORSBOOM D, 2017, WORLD PSYCHIATRY	https://doi.org/10.1002/wps.20375	2496	249.60	29.39
LIVINGSTON G, 2024, LANCET	https://doi.org/10.1016/S0140-6736(24)01296-0	2057	685.67	99.50
MCEWEN BS, 2000, NEUROPSYCHOPHARMACOLOGY	https://doi.org/10.1016/S0893-133X(99)00129-3	1451	53.74	7.03
GUZIK TJ, 2020, CARDIOVASC RES	https://doi.org/10.1093/cvr/cvaa106	1120	160.00	20.11
DANESE A, 2009, ARCH PEDIATR ADOLESC MED	https://doi.org/10.1001/archpediatrics.2009.214	1052	58.44	9.97
CHRISTENSON SA, 2022, LANCET	https://doi.org/10.1016/S0140-6736(22)00470-6	1029	205.80	35.80
CACIOPPO S, 2015, PERSPECT PSYCHOL SCI	https://doi.org/10.1177/1745691615570616	987	82.25	11.41
CACIOPPO JT, 2002, PSYCHOSOM MED	https://doi.org/10.1097/00006842-200205000-00005	929	37.16	5.98
SKOU ST, 2022, NAT REV DISEASE PRIM	https://doi.org/10.1038/s41572-022-00376-4	921	184.20	32.04
SHANKAR A, 2011, HEALTH PSYCHOL	https://doi.org/10.1037/a0022826	851	53.19	9.46

**Fig. 8:**
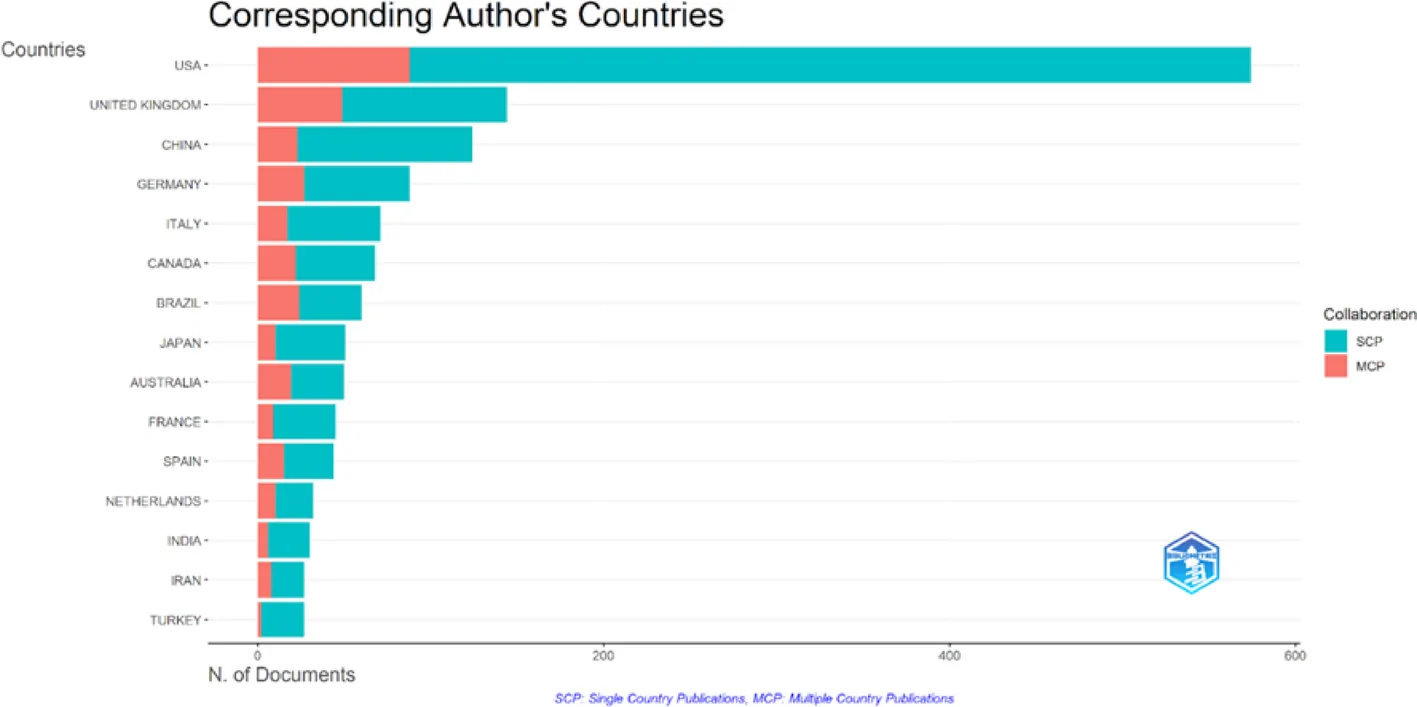
Country-wise production

This Table 2 can be compared with Fig. 4 which projects the most-relevant sources. The most cited papers came from the journals that are not listed in most relevant sources.Hence a key distinction is noted between Fig. 4 and Table 2. The distinction is bibliometrically significant as it reveals the tension between productivity and influence. A source that publishes several papers on the specific topic need not be an influential one; rather, a journal with a highly cited few papers may be intellectually compelling Fig. 8.

Single Country Publications (SCP) represent research activity within one country and Multiple Country Publications (MCP) shows research collaboration between authors from different countries. The figure demonstrates the distribution of publications between authors of SCP and MCP. It reveals that the United States has the substantially highest number of publications, and the majority of them are SCP, though there is a higher level of MCP as well. This indicates the strength of research contribution in their country. UK and China lead next with a balanced output of SCP and MCP, which are followed by Germany and Italy. Canada, Brazil, Japan and Australia contribute an average number of publications including single-country and global collaboration works. Smaller but consistent research publications by France, Spain, and Netherlands put these countries in the international landscape of research. India, Iran and Turkey show relatively modest research contributions.

To sum up, research productivity is concentrated in developed nations along with collaboration among different countries (Tables 3, 4).

**Table 3:** Country-wise total citations

Country	No. of articles	TC
USA	574	37,101
UK	144	13,590
China	124	2243
Germany	88	3062
Italy	71	2043
Canada	68	2942
Brazil	60	1823
Japan	51	1360
Australia	50	1401
France	45	1308
Spain	44	1382
Netherlands	32	1417
India	30	660

**Table 4:** Research activity of Single Country Publications (SCP) and Multiple Country Publications (MCP)

Country	SCP	MCP	Total
USA	486	88	574
UK	95	49	144
China	101	23	124
Germany	61	27	88
Italy	54	17	71
Canada	46	22	68
Brazil	36	24	68
Japan	41	10	51
Australia	31	19	50
France	36	9	45
Spain	29	15	44
Netherlands	22	10	32
India	24	6	30
Iran	19	8	27
Turkey	25	2	27

The performance analysis reveals that the interdisciplinary field has undergone exponential growth in the last decade. This research is dominated by leading global institutions and influential scholars, ensuring high impact and visibility. The presence of highly cited foundational works alongside recent influential studies indicates both conceptual maturity and ongoing innovation.

The bibliometric profile of the dataset reveals important patterns in scientific productivity, collaboration, and geographic distribution of research output.

**Fig. 9:**
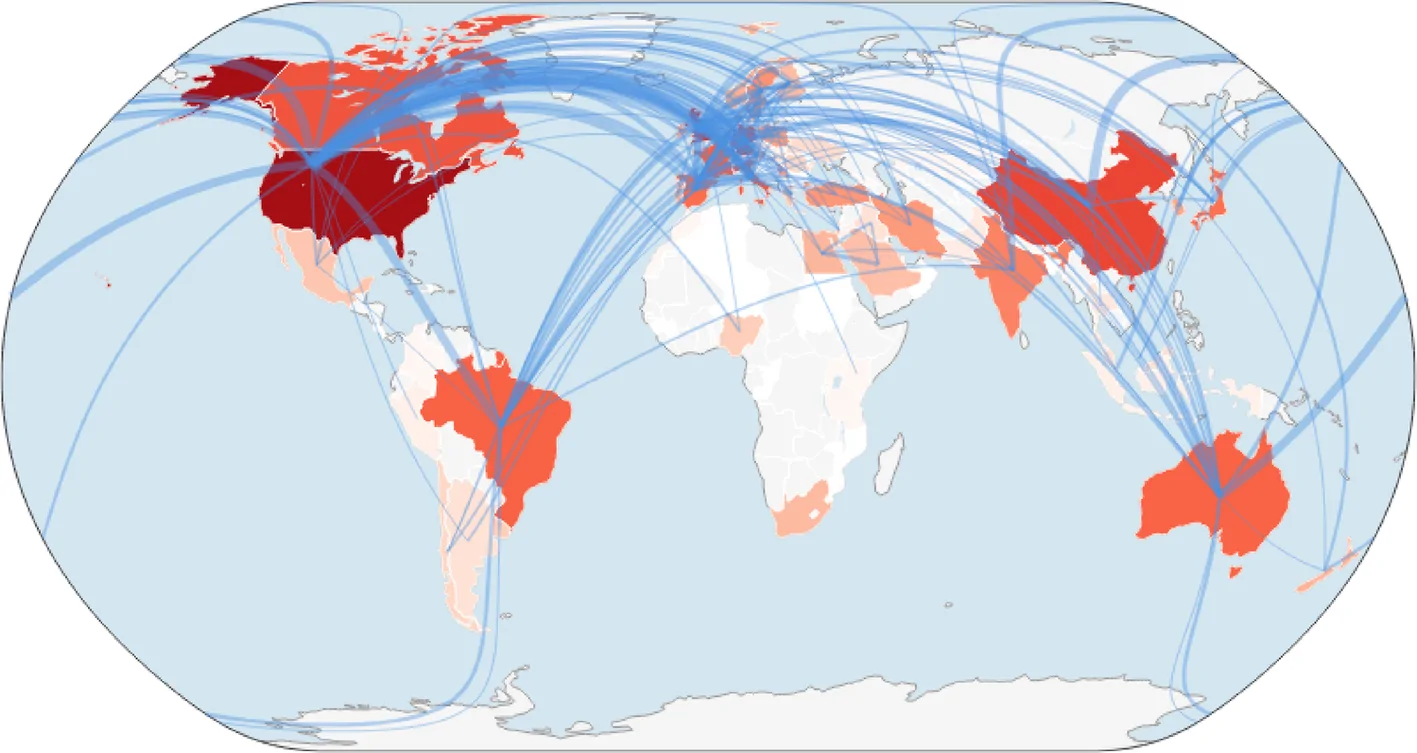
Country-wise collaboration

The Fig. 9 depicts the collaboration network at global level between different countries and co-authors' work. Red colour indicates the level of research contribution. The intensity of the colour denotes the level of research activity with darker shades symbolising higher number of publications. The major hubs of research activity are the United States, China and many European nations. International research collaborative ties are depicted by blue lines. It shows how researchers from different regions work together. North America, Europe and Asia regions demonstrate a highly interconnected research network. Australia, India and Brazil also show notable research productivity. But several regions in Africa and South America have low research activity and less collaborative links. Overall, it can be inferred that developed countries are having major research outputs.

### Country-wise collaborations

Table 5 shows the top countries which have collaborative partnership. US-UK ranks first with 57 publications, followed by US-Canada (35) and US-Germany (33). Further, the UK also shows strong collaborative partnership with Germany (20), Italy (19) and Netherlands (19).

**Table 5:** Number of documents from the top most collaborating countries

Collaborating countries		Frequency
USA	UK	57
USA	Canada	35
USA	Germany	33
UK	Germany	20
USA	China	20
UK	Italy	19
UK	Netherlands	19
UK	Australia	18
USA	Australia	17
USA	Brazil	17

### Science mapping

**Fig. 10:**
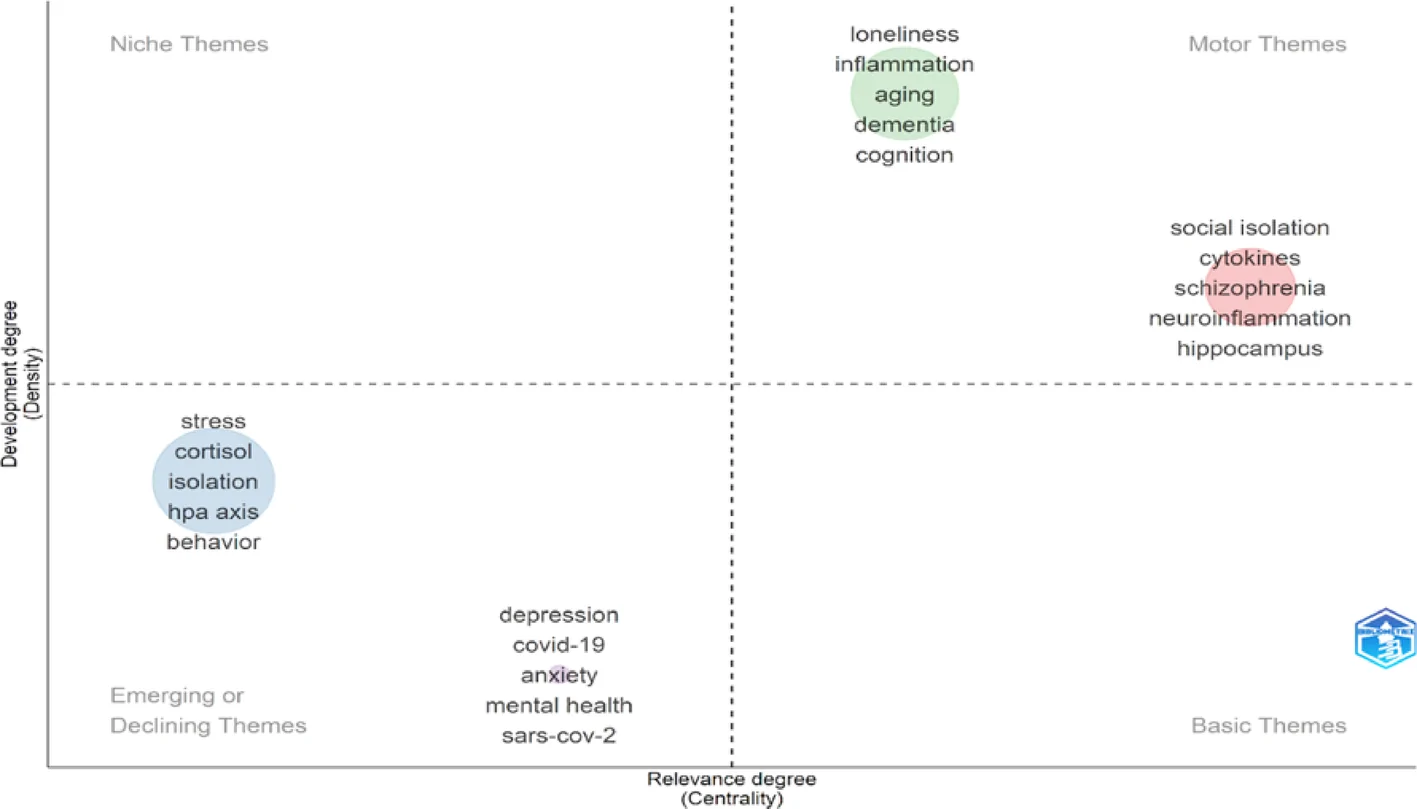
Thematic map

Figure 10 as a thematic map identifies four distinct thematic clusters distributed across four quadrants of centrality and density. This map is divided by the X-axis (Centrality) and Y-axis (Density), forming four quadrants. Centrality indicates the importance of the theme within the broader field, and the density shows how it has been developed over a period of time.

*Motor themes (upper right quadrant)*: This cluster indicates high centrality and density of the themes. This theme suggests loneliness, dementia, social isolation, neuroinflammation, and hippocampus are central drivers in the field linking social factors (isolation) to cognitive ageing and biomarkers.

*Social isolation:* The positioning of social isolation, cytokines, and schizophrenia within the motor-theme quadrant indicates that these topics are both highly developed and strongly connected to the broader knowledge structure of the field, suggesting their central role in contemporary research on the biological consequences of social isolation. Though its density is relatively lesser, it indicates how further in-depth study is required to understand its intricate mechanisms on biological ageing processes.

*Loneliness:* Given the broader landscape of social isolation, loneliness can be mostly understood from a subjective lens, when the individuals feel the lack of connectivity at personal level.

*Dementia:* The presence of dementia as another important theme indicates how social isolation can influence neurobiological mechanisms resulting in cognitive decline over a period of time.

*Ageing:* This theme has emerged as one of the important research areas due to the practical implications of social isolation on acceleration of biological processes. Its presence as one strong theme underscores the association with loneliness, inflammation and cognitive decline.

*Inflammation:* Terms like inflammation and neuroinflammation indicate how social connectivity would influence molecular processes of ageing. It suggests the relationship between physiological pathways and chronic inflammation leading to faster biological ageing of individuals.

Niche themes (upper left quadrant): This theme is characterized by high density (strong internal cohesion and specialised topic) but low centrality (weak external connections to other field areas). It has not yet been fully integrated into the field’s core topics.

Emerging or declining themes (lower left quadrant): This specific theme represents research areas that are underdeveloped, marginal, and emerging in the current structure of the field. The cluster around stress, cortisol, isolation, HPA axis, and behaviour looks more like an emerging or consolidating research area. The cluster of depression, covid 19, anxiety, and mental health is relevant as this is closely tied with global health concerns. It reflects a nexus between the pandemic, increased levels of anxiety, depression, and mental-health issues, which have become a central concern in public health and social science research.

Basic themes: The basic themes quadrant represents that the field seems to be more organised around well-established themes. The current research trend in social isolation and biomarkers of ageing is focused on core themes like neurobiological processes, inflammatory pathways, and stress etc. The clustering technique and minimal keyword frequency criterion restrict the formation of smaller theme groups, as it lacks the statistical thresholds necessary to reflect in this quadrant.

They often point at future growth directions linked to new technologies, policy shifts, or theoretical innovations. This indicates that this field has only a small number of publications, weak internal cohesion, and few links to core or motor themes, placing it away from the core intellectual structure. It may be interpreted as a nascent topic with growth potential, especially if recent years show a rising publication trend and tentative links to broader debates; interpreted as declining, it may reflect a once-active area that is now losing momentum, citations, and conceptual relevance as the field shifts toward other priorities. In either case, the lower-left position highlights an area that is not yet (or no longer) integrated into the mainstream and thus offers opportunities for either strategic investment or critical reflection on why it remains marginal.

However, clustering and visualization algorithms render it as an isolated “island” of specialized work.

**Fig. 11:**
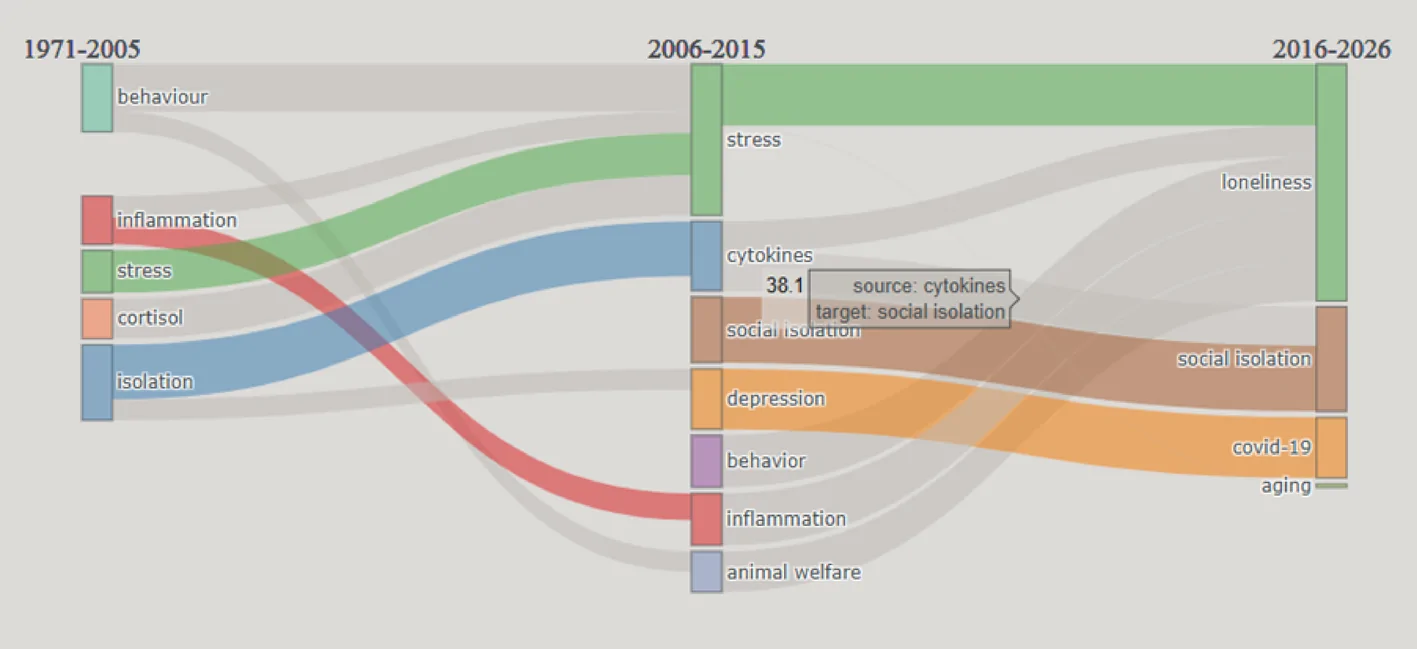
Thematic evolution

Figure 11 illustrates the temporal dynamics of keyword clusters during 1971–2005, 2006–2015 and 2016–2026.

The width of the flow suggests how shared keywords and connections between themes vary across different time slices. It provides insights into intellectual structure, dominant research trajectories, emerging themes in those different periods.

To examine the temporal evolution of research themes, the study period was divided into three phases based on publication growth patterns observed in the annual scientific production curve: 1971–2005 (foundational phase), 2006–2015 (development phase), and 2016–2026 (expansion phase). The first phase was characterized by relatively low and sporadic publication activity, reflecting the formative stage of research on social isolation and biological markers.The field began with biologically-anchored vocabulary such as behaviour, inflammation, stress, cortisol and isolation. The second phase witnessed a gradual increase in scientific output and thematic diversification. The new themes emerged in the second phase are cytokines, social isolation, depression, behaviour, inflammation and animal welfare. The third phase was marked by a substantial growth in publications after 2015, indicating increased scholarly attention to the biological implications of social isolation and the emergence of more specialized research themes such as loneliness, social isolation, covid 19 and ageing. The thematic overlap index (38.1) between cytokines and social isolation suggests these constructs were frequently investigated together.

**Fig. 12:**
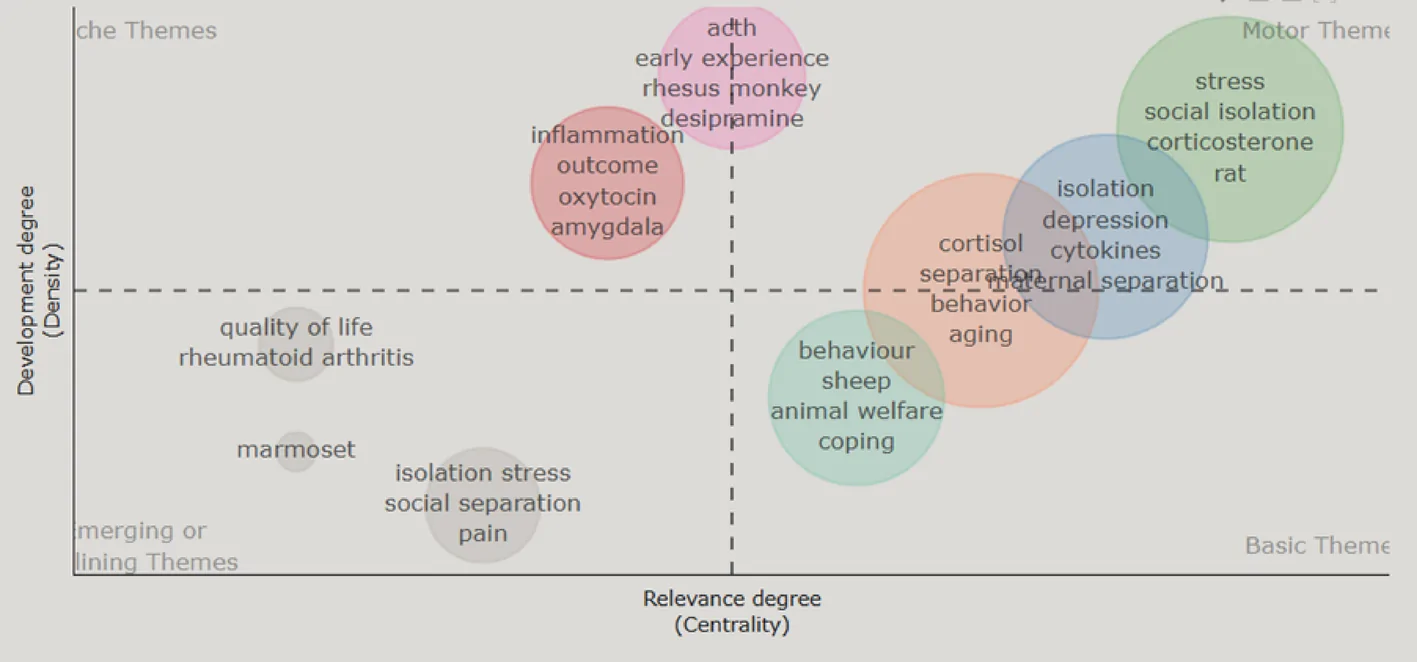
Thematic evolution (1971–2005)

The thematic focus of Fig. 12 is from 1971 to 2005.

The research themes are primarily focused on behaviour, inflammation, cortisol, and isolation. The map depicts the biological stress responses and behavioural aspects in relation to social isolation. This field of research is gradually evolving from specialised biological studies to a more embedded or integrative approach.

**Fig. 13:**
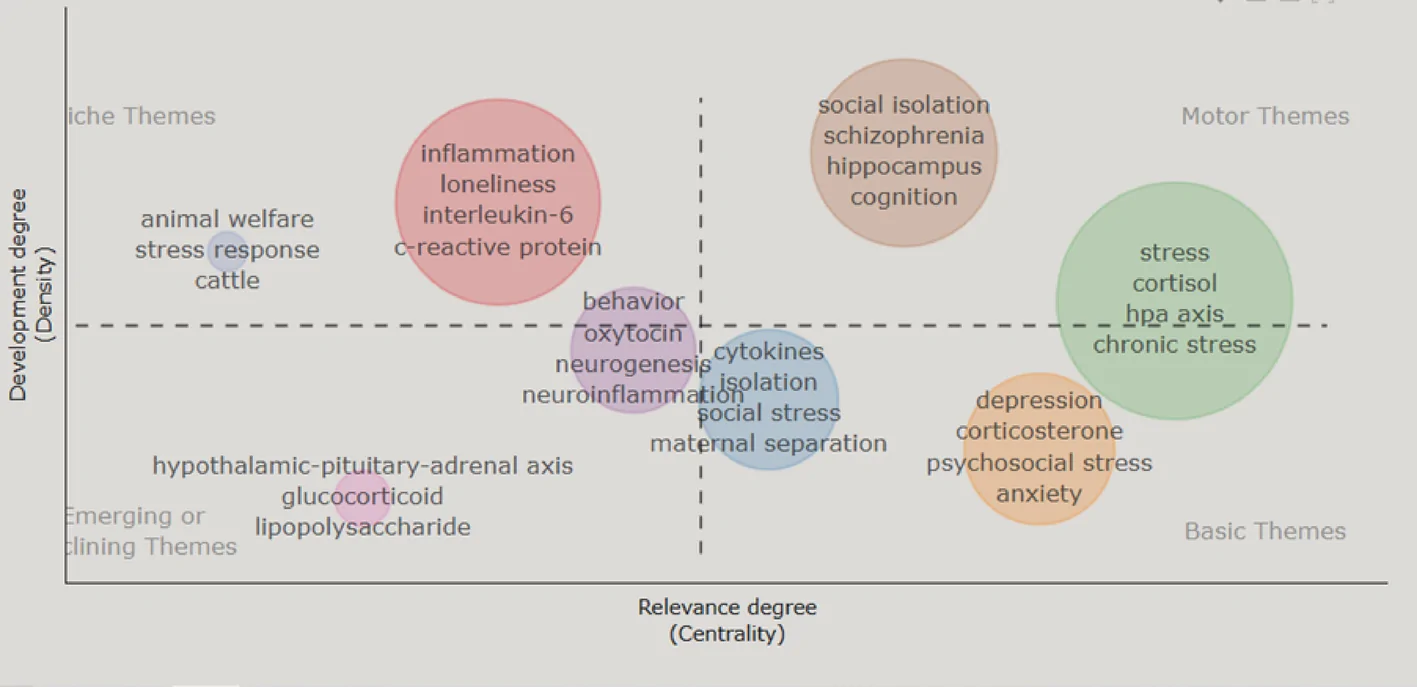
Thematic evolution (2006–2015)

Figure 13 represents the core research areas from 2006 to 2015.

The core cluster of the field focuses on the biological stress-response, such as the HPA axis, oxytocin, cytokines, neuroinflammation, depression, corticosterone, C-reactive protein, interleukin-6. It indicates advances in psychological consequences as well as biological processes.

**Fig. 14:**
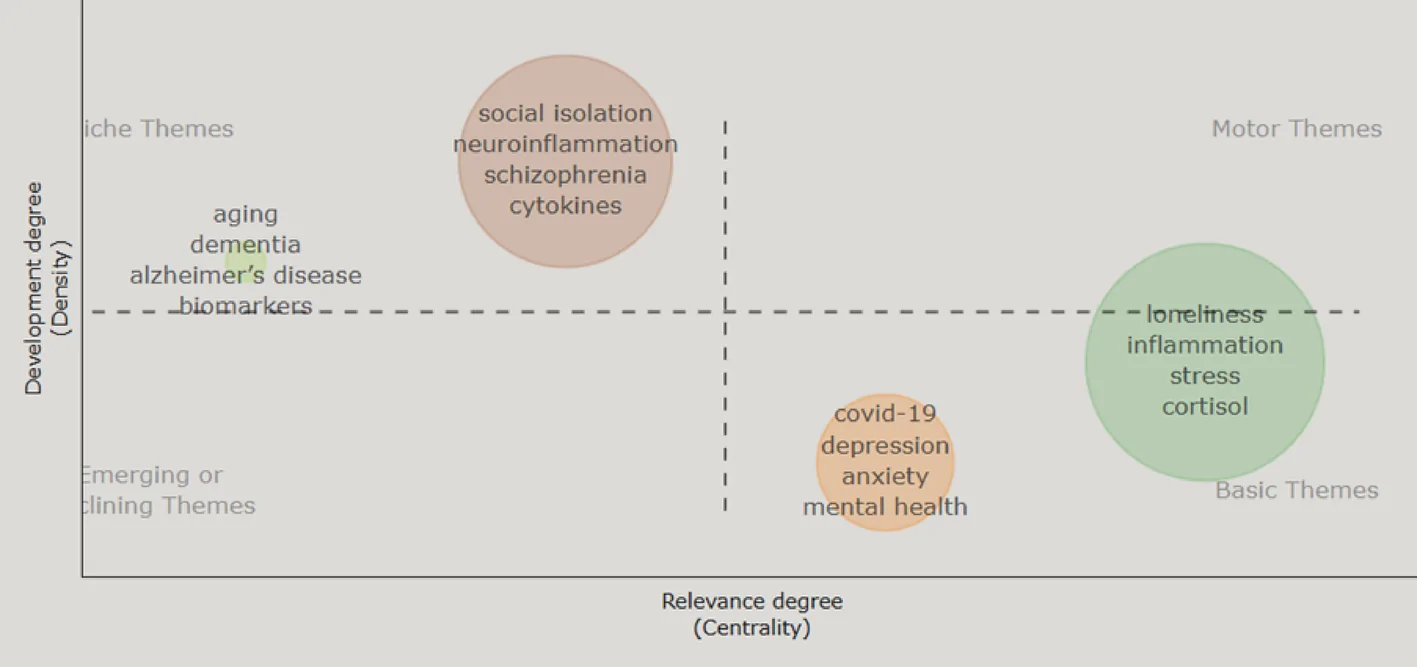
Thematic evolution (2016–2026)

Figure 14 displays the predominant themes such as loneliness, social isolation, COVID-19, and ageing during the period (2016–2026).

Their trend depicts the integration of psychological experiences and biological mechanisms. The themes of COVID-19 examine the social isolation experience on the well-being of individuals. The presence of stress, cytokines, and social isolation along with age-related health issues, shows the importance of biomarkers of ageing.

Therefore, the thematic evolution shows how the field has evolved from isolated behavioral studies, ageing studies, to multidisciplinary frameworks involving neurobiological processes, psychosocial factors and in general mental and physical well-being of individuals. The early phase focused on basic biological and behavioural responses like stress, cortisol, molecular level changes. The development phase focused on more complex psychological factors like depression, social isolation, and immune system indicators like cytokines. In the latest periods the research is studying more about public health concerns, demographic factors like ageing, in context like covid-19 as well.

**Fig. 15:**
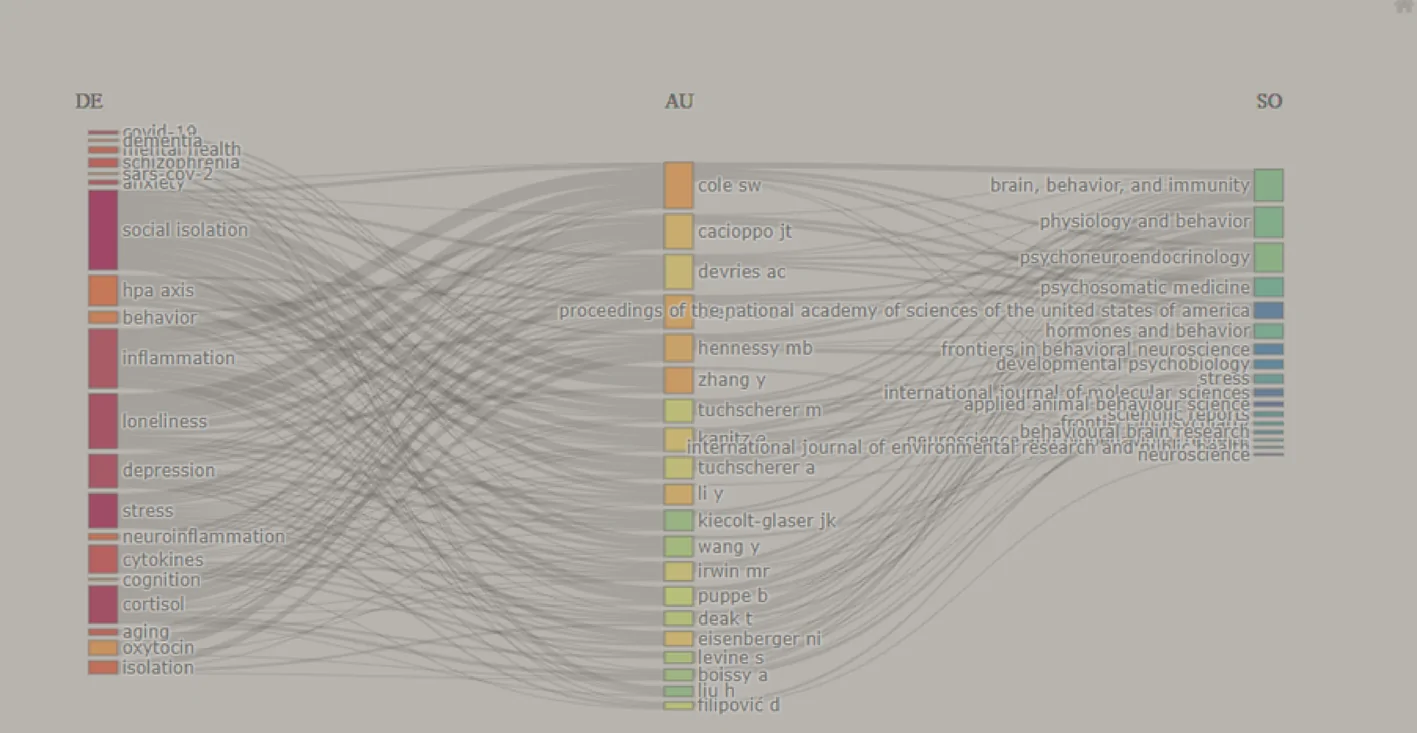
Three-field plot

Figure 15 reveals the interrelationship between keywords, authors and source journals. DE (themes of research) stands for research topics, AU (middle column) for Authors, and SO refers to sources. The curved lines represent the flow of the knowledge. It connects how the topics are connected to authors and where these authors publish these in journals. The thickness of the connecting lines shows the strength of the relationship. The colour represents the keyword clusters. The keywords such as inflammation, cytokines, neuroinflammation, cortisol, oxytocin show a strong biomedical orientation.

The broad range of themes such as loneliness, social isolation, and resilience are, contributing to multiple high impact journals. Among the authors in this field, is Cacioppo JT is pivotal in linking multiple descriptors and publishing in high-impact journals. Cole SW is a key contributor to social isolation, inflammation,gene expression pathway publishing in *Brain, Behaviour, and Immunity*. Kiecolt-Glaser JK and Irwin MR are key contributing authors, reinforcing the psychosomatic and stress-related research streams. Authors such as Zhang Y, Li Y indicate diversification into molecular sciences during the COVID-19 era. Journals such as *Brain, Behavior, and Immunity; Psychology and Behaviour; Psychoneuroendocrinology; and Psychosomatic Medicine* dominate the field of research as primary publishers for interdisciplinary works, reflects psychoneuroendocrinological and biomedical orientation of this field, consistent with the thematic map findings. However, the three field plot underscores that the literature on social isolation and biomarkers of ageing is secured to a small number of highly influential scholars.

### Factorial analysis

**Fig. 16:**
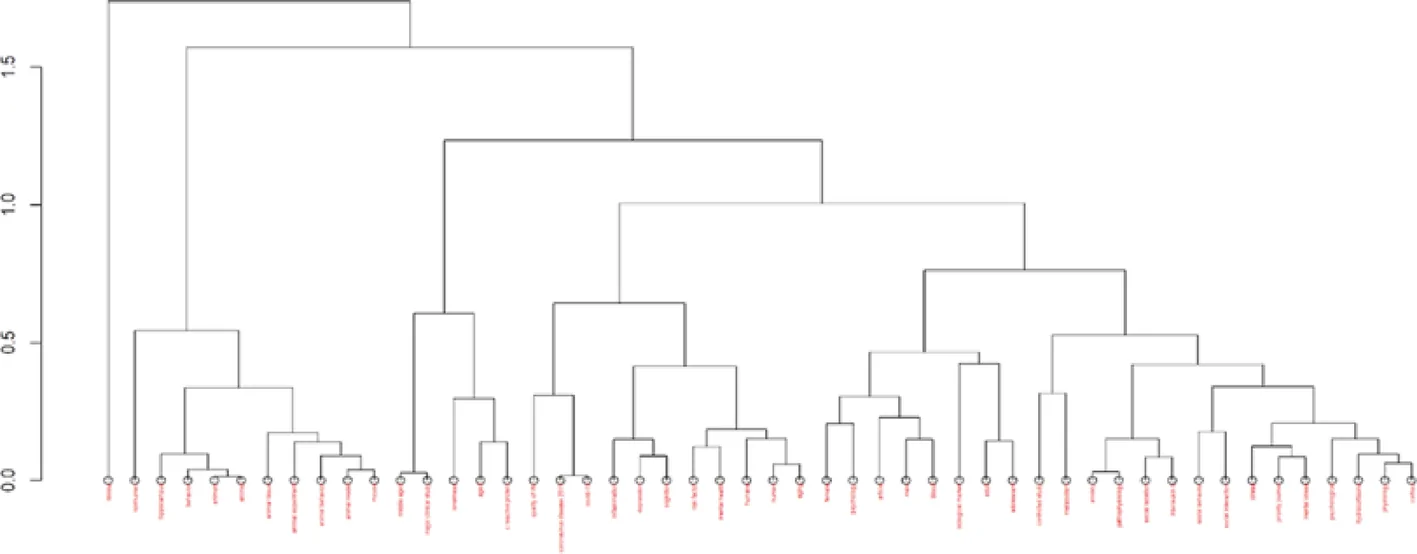
Dendogram

Figure 16 is a dendrogram. It presents a hierarchical clustering of the 50 keywords that co-occur together in the literature. There are around 5 major clusters out of the total 12 clusters. Cluster 1 (far right) indicates the most distinct cluster from the rest of the field. Keywords noted here include non-human, animal, behaviour, hippocampus, mouse, animal experiments.This cluster is on the preclinical and animal model literature. Cluster 2 talks about the clinical disease and inflammation cluster (Left-Center) with the keywords such as loneliness, ageing, quality of life, C-reactive protein, COVID-19, inflammation, depression, cognition, risk factor, mental health, and human. This cluster represents human clinical epidemiology. Studies linking social isolation to inflammatory biomarkers, mental health outcomes, cognitive decline, and disease risk have been found. The presence of COVID-19-related terms here is significant, reflecting the surge of pandemic-era research on social isolation and health consequences. Cluster 3 is about ageing, biology, and psychophysiology cluster (Centre-Right). This cluster includes reference to female, age, psychobiology, brain, biological markers, adolescent, controlled study, and metabolism. This represents research on the biological mechanisms of ageing including sex differences, metabolic markers, neurobiological correlates, and developmental perspectives. Cluster 4, the rightmost one, is the psychosocial and intervention cluster, containing keywords such as social isolation, loneliness, social behavior, mental disorder, priority journal, psychological, neuropsychology, and physiological.

These studies have focused on the subjective experience of isolation, its psychological dimensions, and its relationship to mental and physiological health outcomes. The relatively high merge heights of the cluster suggest that this field is still intellectually fragmented. The finding worth flagging is a conceptual measurement problem which is evident from the close sub-clustering of social isolation, loneliness, and psychological terms on the far right at very low dissimilarity values. This indicates these concepts are used interchangeably in a subset of the literature.

Multiple Correspondence Analysis (MCA) was conducted using all keywords, considering the 50 most frequent terms to explore the conceptual structure of the field and visualise relationships among dominant research themes.

**Fig. 17:**
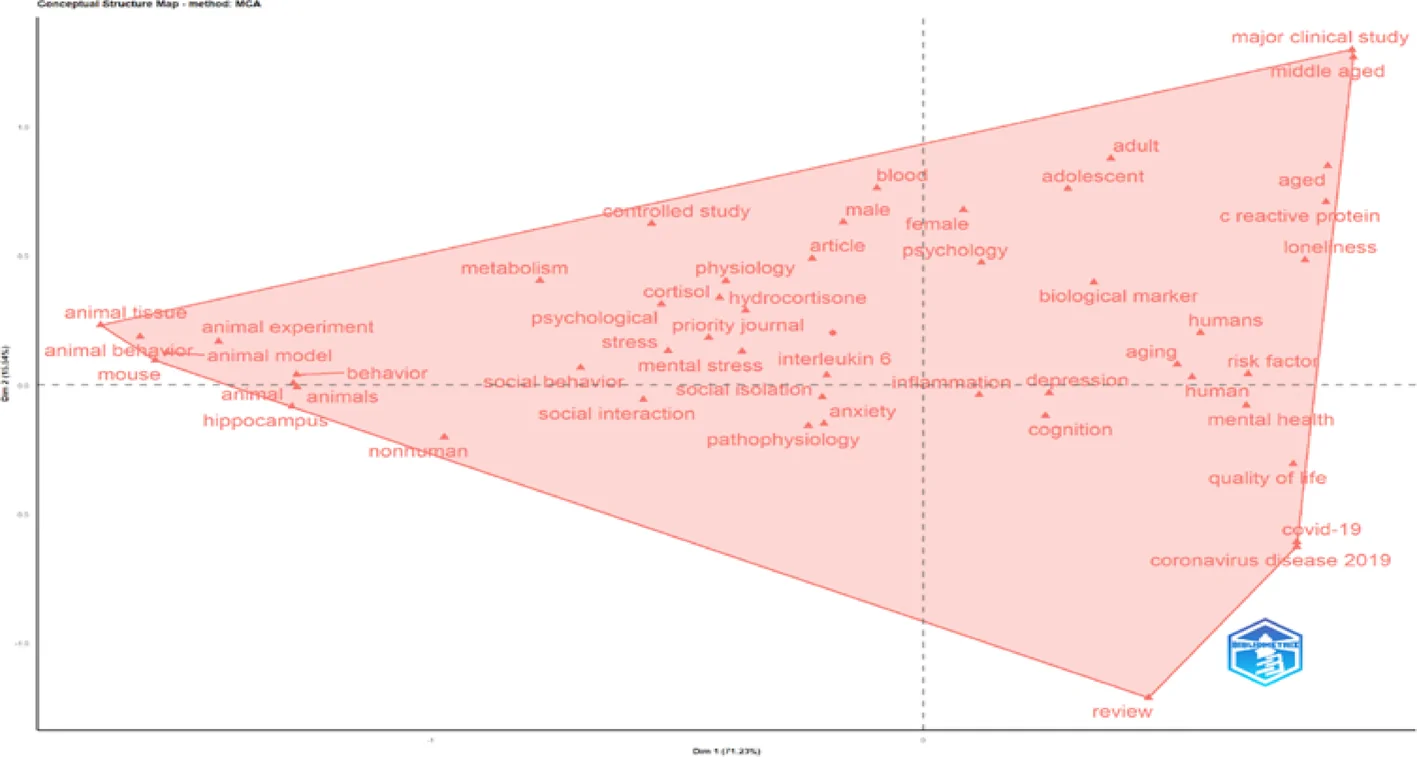
Factorial map

Figure 17 identifies two principal factorial dimensions. It explains the conceptual variations across 1973 publications. The factorial X axis (horizontal) contrasts preclinical animal based research against human clinical research. The trajectory reflects the advancement of the field from laboratory investigation to human level inquiry into the biological consequences of social isolation. The factorial Y axis (vertical) separates observational descriptive studies (blood, psychology, male, female, major clinical study) from mechanistic developmental studies (hippocampus, pathophysiology and social interaction). It means within human research tradition some studies observe and describe while others examine the mechanisms and developmental pathways.

However, the Multiple Correspondence Analysis (MCA) revealed a highly interconnected conceptual structure within the literature on social isolation and biomarkers of ageing. Keywords related to psychosocial experiences (e.g., loneliness, social isolation, anxiety, depression, and mental health) were positioned in close proximity to biological and physiological concepts such as inflammation, interleukin-6, cortisol, C-reactive protein, and biological markers. Ageing-related terms (e.g., ageing, aged, and adult) and experimental research concepts (e.g., animal model, mouse, and hippocampus) were also integrated within the same conceptual space. The absence of distinct clusters suggests that research on social isolation and biomarkers of ageing has evolved as a cohesive and interdisciplinary field that integrates psychosocial, biological, clinical, and ageing perspectives.

## Discussion

The present study provides an overall framework of social isolation and biomarkers of the ageing research landscape. It highlights the global collaborations and traces the emerging research trends.

The dashboard of bibliometric analysis spans 1971–2026 (55 years) and documents 1973 papers from 907 sources. The field has grown steadily with an annual growth rate of 7.76 percent. 9758 authors have contributed to the field. This field of research confirms that collaboration is the norm, especially team effort, not solo authorship. International collaboration is a moderate trend, with about 1 in 4 papers involving authors from more than one country. The average citation per document is 50.56, which suggests the documents are influential in their fields. The most cited work is not very old. The increase in publication trajectory noticed in the current study shows the importance of sustained growth in studies related to social isolation. Also, the bibliometric analysis should reflect expansion of research activity and the role of international collaboration [35].

The journals that publish these articles characterise it as a multidisciplinary field that intersects psychology, neurobiology, endocrinology, immunology, neurology and psychiatry. The leading journals in this field are *Psychoneuroendocrinology* leads with 74 publications, followed by *Brain, Behavior, and Immunity* (57), *Physiology and Behavior* (55). The scholarly works by Cole SW (25 publications), Zhang Y (20 publications), Steptoe A (17 publications), Hennessy MB and Li Y (16 each) and Cacioppo JT, Eisenberger NI, and Kanitz E (14 each) are noteworthy.

A cross-reference comparison between Table 2 (most cited papers) and Fig. 2 (most relevant sources) unveils that though Psychoneuroendocrinology, Brain, Behaviour and Immunity; and Physiology and Behaviour publish often and steadily but they are not where the single most influential highly cited papers came from. Instead the most cited papers tend to appear in journals like World psychiatry, Lancet and Neuropsychopharmalogy that publish fewer papers but achieve high citation impact. Of the ten most cited documents, only one Psychosomatic Medicine (ranked 6th in productivity with 28 documents) appeared among ten most relevant sources.

The institutions that play a significant role in creating strong research hubs are. University of California (139), followed by University College London (53), University of Chicago (44), The Ohio State University (40) and King’s College London (38).It shows the western dominance in this field of research.

The top ten cited research papers in the domain of social isolation and the biomarkers of ageing offer a deep insight into this multifaceted field. Collectively, these seminal papers have advanced the knowledge in the field of social isolation and the biomarkers of ageing linking loneliness and psychological stress to disease risk and premature ageing uniquely, contributing to the discourse of social isolation and the biomarkers of ageing. They show that social isolation and loneliness act as a chronic stressor that elevates cardiometabolic, inflammatory, and neuroendocrine biomarkers (pro-inflammatory cytokine, cortisol and allostatic load), which finally end up with age-related disease, dementia, and earlier mortality.

The USA is the leading country in terms of the research contribution to this knowledge domain followed by the UK and China. Next comes Germany and Italy. Canada, Brazil, Japan and Australia contribute an average number of publications including single-country and global collaboration works. France, Spain, and the Netherlands put these countries in the international landscape of research in terms of smaller but consistent research publications. India, Iran and Turkey have maintained modest research contributions. To sum up, research productivity is concentrated in developed nations along with collaboration among different countries. A study by Ong et al. [36] identified major contributors and emerging themes through bibliometric mapping of spinal cord injury and rehabilitation research. Similarly the present study highlights top countries and their most cited documents in the research of social isolation and biomarkers of ageing.

The bibliometric techniques can be used to map thematic clusters and new research trends, similar to the mapping of intellectual structure through network analysis [37]. The thematic map or quadrant structure suggests that the current theme of research is anchored in a robust core area such as loneliness, dementia, social isolation, neuroinflammation, and hippocampus, confirming that they are the central driver of the domain (Scientific Report 2024). The analysis reveals that the niche and emerging themes are not fully integrated with the core areas. The topics such as stress, cortisol, isolation, HPA axis, and behaviour looks more like an emerging or consolidating research area [12]. Covid 19 in niche theme points at pandemic research’s technical isolation but weak ageing integration [13]. However, this domain seems to be more organised around well-established themes. The findings from other bibliometric mappings of geriatric research domain, such as falls in old adults [38] where similarly fragmented but connected thematic clusters emerged around physiological, behavioural, and environmental risk factors, are consistent with this pattern of thematic clustering around biological and behavioural risk factors in ageing populations.

The shape of the MCA map which is a cone that narrows to the left and widens to the right, is an important finding. It underscores how the field started with narrowly focused animal experimentation and has gradually grown into a varied expansive conceptual landscape that includes multi-biomarker human research, clinical trials, and epidemiological studies. The development of the field from mechanistic laboratory research to human-level exploration into the biological effects of social isolation throughout life is demonstrated in this trajectory. The integration of social disconnection and biological ageing is the current frontier of this research domain, as evidenced by the placement of loneliness, biological marker, ageing, and cognition in the far-right zone, which is the most conceptually advanced region.

The bibliometric analysis of social isolation and biomarkers of ageing gives important insights with crucial inferences for psychosocial research of biological ageing. By finding key research publications, authors, trends of themes, evolution of new themes, this study emphasizes the role of social network, community life and relationships of individuals on their neurobiological processes resulting in acceleration of physiological ageing. The research gives valuable references of research contributions of influential authors, journals, institutions, and potential collaborators and publications. This study helps to understand how social isolation affects mental and physical health which can be used to develop evidence-based interventions targeting the vulnerable population. The collaboration between countries shows how most of the research activity is concentrated in developed countries. Such collaboration can be expanded to other moderate and underdeveloped countries to produce more diverse findings.

## Future research propositions

Based on the above-mentioned results emerging research clusters are identified which highlight some potential future areas of study. The following clusters can provide directions in an interdisciplinary perspective. They have been derived from conceptual analysis of literature related to social isolation and biomarkers of ageing.

Cluster 1: Social isolation and inflammatory responses

This cluster examines the connection between social isolation and specific biomarkers which are inflammatory in nature, namely cytokines, interleukins, and C-reactive protein. These inflammatory biomarkers can be triggered by social isolation and loneliness which in turn causes several ill-health outcomes like risk of cardiovascular diseases [39]. Another important study by Cudjoe et al. [12] found that though there is no clear biological mechanism that connects social isolation with morbidity, Interleukin- 6 (IL-6) and C-reactive protein (CRP) can result in dementia and cognitive decline in individuals as they age. Hence more studies to understand the causal pathways of inflammation and its link with psychosocial stressors are needed.

Research proposition 1a: How inflammatory biomarkers are influenced by social isolation across adulthood stages?

Research proposition 1b: Can social connectivity reduce inflammatory responses?

Cluster 2: Physiology of stress and biomarkers of hormones

This cluster focuses on the role of cortisol and other stress-related biomarkers. Though cortisol and hypothalamic–pituitary–adrenal (HAP axis) have long been isolated with physiological stressors, long periods of psychological stress also would trigger the similar activity of hormones. In a longitudinal study of National Survey of Health and Development (Britain) conducted by Stafford et al. [40] found that despite of multiple correlates of behavioral and psychosocial factors, recent onset of social isolation increased diurnal cortisol patterns which further increased the risk of morbidity and mortality.

Research proposition 2a: Whether cortisol regulation and stress hormone levels are dependent on factors like gender, lifestyle socio-economic status?

Research proposition 2b: How social isolation is culturally defined across different geographic regions to understand its influence on stress-related physiological responses?

Cluster 3: Epigenetic ageing and epigenetic clock

This is another important area which discusses epigenetic indicators like telomere length, DNA methylation and epigenetic clocks. Since epigenetic processes are also associated with environmental triggers, these markers would provide insights into how psychosocial factors would accelerate or decelerate biological ageing. A study conducted by Vaiserman and Krasnienkov [41] found that social disconnection can impact cellular senescence, leading to accelerated rate of telomere attrition. Loneliness can produce hyper sympathetic responses and lower parasympathetic activity, for example heart rate variability (HRV) resulting in activation of cytomegalovirus virus, causing shorter telomere length [42].

Research proposition 3a: How exactly can social isolation influence epigenetic processes?

Research proposition 3b: How to measure the sensitivity of epigenetic indicators when triggered by psychosocial stressors?

## Conclusion

However, the bibliometric study is fundamentally a quantitative study which cannot assess the qualitative aspects of the research work. As this bibliometric study is confined to a single bibliometric database, it has omitted relevant studies indexed in PubMed and other regional databases. Additionally, the keywords used in the search string are not standardized across journals. Different authors may have used different terminologies to describe the concept. The articles published earlier in the study period may have more time to accrue citations than recent works. Consequently, the critical research works published within the last few years may appear less influential. In the wake of the COVID-19 pandemic, this field of social isolation and biomarkers of ageing has gained a temporal surge with new publications. Following this, it raises the question “to what extent can social isolation be attributed to Covid-19 induced isolation or institutional transformations in family, marriage and community life”.

Hence, there are several potential areas of research to further discover the biopsychosocial intersections between mental health, social isolation and physiological health. The future studies can attempt at understanding the complexities within the biomarkers. Few biomarkers like interleukin (IL-6), epigenetic clocks are indicators of other physiological processes as well. The association between mental health conditions like depression, anxiety, the mediating role of psychosocial stressors and the overall physical health of a person remain relatively vague. Also some socially disconnected or lonely individuals show accelerated biological ageing while others remain relatively resilient. This research can be further used to understand how far these biomedical and psychological factors are instrumental in different stages of life to design intervention models. The intervention and prevention-oriented study of social isolation can be made part of community-based programs, psychosocial interventions to enhance social connectivity and act as a buffer for biological stress. The digital platforms and technologies can be constructed with concepts of social connectedness and neurobiology of stress models, particularly in ageing and low-resource settings.

## Data Availability

No datasets were generated or analysed during the current study.
